# Prevention of Diet-Induced Obesity Effects on Body Weight and Gut Microbiota in Mice Treated Chronically with Δ^9^-Tetrahydrocannabinol

**DOI:** 10.1371/journal.pone.0144270

**Published:** 2015-12-03

**Authors:** Nina L. Cluny, Catherine M. Keenan, Raylene A. Reimer, Bernard Le Foll, Keith A. Sharkey

**Affiliations:** 1 Hotchkiss Brain Institute and Snyder Institute of Chronic Diseases, Department of Physiology and Pharmacology, University of Calgary, Calgary, Alberta, Canada; 2 Faculty of Kinesiology and Department of Biochemistry and Molecular Biology, University of Calgary, Calgary, Alberta, Canada; 3 Translational Addiction Research Laboratory, Campbell Family Mental Health Research Institute, Centre for Addiction and Mental Health (CAMH), Toronto, Ontario, Canada; University of Minnesota, UNITED STATES

## Abstract

**Objective:**

Acute administration of cannabinoid CB_1_ receptor agonists, or the ingestion of cannabis, induces short-term hyperphagia. However, the incidence of obesity is lower in frequent cannabis users compared to non-users. Gut microbiota affects host metabolism and altered microbial profiles are observed in obese states. Gut microbiota modifies adipogenesis through actions on the endocannabinoid system. This study investigated the effect of chronic THC administration on body weight and gut microbiota in diet-induced obese (DIO) and lean mice.

**Methods:**

Adult male DIO and lean mice were treated daily with vehicle or THC (2mg/kg for 3 weeks and 4 mg/kg for 1 additional week). Body weight, fat mass, energy intake, locomotor activity, whole gut transit and gut microbiota were measured longitudinally.

**Results:**

THC reduced weight gain, fat mass gain and energy intake in DIO but not lean mice. DIO-induced changes in select gut microbiota were prevented in mice chronically administered THC. THC had no effect on locomotor activity or whole gut transit in either lean or DIO mice.

**Conclusions:**

Chronic THC treatment reduced energy intake and prevented high fat diet-induced increases in body weight and adiposity; effects that were unlikely to be a result of sedation or altered gastrointestinal transit. Changes in gut microbiota potentially contribute to chronic THC-induced actions on body weight in obesity.

## Introduction

Obesity has reached epidemic proportions worldwide placing a huge health and economic burden on society. Lifestyle modifications such as improved diet and increased physical activity can initially be successful in reducing weight, but maintaining weight loss through these measures proves difficult [1]. Indeed, only surgical approaches to reverse or limit weight gain have proven successful in the long term [2].

It is well established that the endocannabinoid system is involved in both the regulation of energy balance and the development of obesity. Cannabinoid (CB)_1_ receptor deficient mice are lean and are resistant to diet-induced obesity when fed a high-fat diet [3]. CB_1_ receptors are located in many regions involved in the control of body weight such as the hypothalamus, limbic system, gastrointestinal tract, adipose tissue, pancreas and liver [4]. Cannabinoid receptor antagonists/inverse agonists [5, 6] and neutral antagonists [7–9] inhibit food intake. And conversely, CB_1_ receptor activation by agonists stimulates short-term feeding when administered acutely [10]. Furthermore, endocannabinoid tone is altered in obesity and with high fat feeding [11–14]. Smoking cannabis is associated with increased short-term energy intake. The major psychoactive constituent of cannabis, Δ^9^ tetrahydrocannabinol (THC) increases food intake and synthetic THC, Dronabinol®, is prescribed as an appetite-stimulant in HIV-related anorexia [15]. However, contrary to what might be expected, the body mass index of regular cannabis smokers was lower than that of non-users [16].

The gut microbiota is an ecosystem containing trillions of bacteria in the distal gastrointestinal tract. It was first shown in 2004 that the gut microbiota is capable of altering host body weight; germ free mice (so called because they are raised free of microorganisms) are lean, and when transfected with microbiota from conventionally-raised mice, they gain weight despite lower food intake and higher energy expenditure [17]. Microbiota have been shown to play a role in the development of diet-induced obesity through the observation that germ-free mice are resistant to the obesogenic effects of a high fat, high sugar diet [18]. These mice have increased fatty acid oxidation in skeletal muscle and liver, and show reduced lipoprotein lipase activity and fatty acid storage in adipocytes, due to increased levels of fasting-induced adipose factor (Fiaf) [18]

Through dietary interventions it has been shown that alterations to the gut microbiota can modify gut hormone production and reduce food intake, leading to weight loss [19]. Furthermore, gut microbiota are involved in the regulation of adipogenesis through endocannabinoid signaling, and changes in microbiota (i.e. prebiotic treatment, antibiotics and germ free conditions) can reverse obesity-induced changes in endocannabinoid tone in adipose tissue [20].

To follow up on our hypothesis that exposure to THC may produce weight loss [21], in the current study we investigated whether chronic THC inhibits weight gain in lean and diet-induced obese (DIO) mice. Due to evidence that the gut microbiota regulates endocannabinoid tone in both the GI tract and adipose tissue [20] we also investigated the effect of chronic THC administration on the gut microbiota.

## Materials and Methods

### Animals and diets

Male C57BL/6N mice (Charles River; Montreal, Quebec, Canada) aged 3–4 weeks on arrival, were group housed (up to 4 mice per cage), under a 12 h light–dark cycle (lights off 19:00) in plastic sawdust floor cages and allowed free access to tap water. Mice were randomly assigned to be *ad libitum* fed either a low-fat diet (n = 16; body weight 20.5 ± 0.3 g; Pico-Vac Lab Rodent Diet #5061, 13.2% kcal from fat, Lab Diet, St. Louis, MO, USA) or a high fat diet (HFD; n = 16; body weight 20.7 ± 0.3 g; D12451, 45% kcal from fat, Research Diets Inc., New Brunswick, NJ, USA) for 6 weeks. Body weight of each mouse, and food intake of each cage, was measured 3 times per week. Food intake (g) was calculated per mouse and converted to energy intake (kcal). This study was approved by the University of Calgary Animal Care Committee (protocol number: M11012) and experimental protocols in this study were carried out in accordance with the guidelines of the Canadian Council on Animal Care. Mice were euthanized at the end of the study by isoflurane overdose.

### Treatments

Δ^9^-tetrahydrocannabinol (THC; Lipomed AG, Arlesheim, Switzerland) was dissolved in a vehicle of ethanol:Tween80:saline at 1:1:98 for the administration of a final dose of 2 mg/kg, and 2:1:97 for the administration of a final dose of 4 mg/kg. THC and corresponding vehicle injections were administered intraperitoneally in a volume of 4 μl/g body weight. Diet-induced obese (DIO) HFD fed mice were randomly assigned to vehicle (n = 7; body weight 29.7 ± 1.0 g) or THC (n = 8; body weight 29.3 ± 0.7 g) treatment groups. Lean mice fed a low-fat diet were randomly assigned to vehicle (n = 6; body weight 24.7 ± 0.5 g) or THC (n = 5; body weight 23.8 ± 0.5 g) treatment groups. Vehicle or 2 mg/kg THC was administered daily (at 16:30, except for locomotor activity studies where treatments were administered between 13:00 and 16:00 on the test day) for 21 days followed by vehicle or 4 mg/kg THC for a further 7 days. Our dosing schedule was based on consideration of the adverse centrally-mediated effects attributed to chronic administration of THC. Suppressed locomotor activity by acute THC has been observed at doses as low as 3 mg/kg in mice [22]. Within days, daily administration of 10 mg/kg THC in rats induced CNS-inhibition (ataxia, impaired coordination and reduced exploratory behavior); tolerance developed to these effects and by the second week of treatment parameters indicative of CNS-stimulation were observed, including hypersensitivity and aggression [23]. Mice administered daily doses of 5 mg/kg THC also became aggressive and easily startled [24]. In an attempt to avoid the development of such aversive effects, a daily dose of 2 mg/kg THC was utilized in the study. After 3 weeks of daily 2 mg/kg THC treatment the dose was increased to 4 mg/kg THC daily, for one week, to investigate the effects of a higher dose.

### Body composition

Duplicate measurements of fat mass were collected from unanaesthetized mice once a week during the treatment period using a Minispec LF110 (Bruker Optics, Billerica, MA), a nuclear magnetic resonance analyzer. Mice were placed individually in a plastic cylinder. Mice were able to move and turn around but the insertion of a plastic plunger into the cylinder restricted their lateral movements to 6 cm. The cylinder was placed in the chamber of the Minispec where duplicate measurements of body composition were calculated. Mice were inside the instrument for approximately 4 minutes before being returned to the home cage.

### Locomotor activity studies

Ambulatory locomotor activity was recorded using an infrared beam activity monitor (Columbus Instruments, Columbus, OH). Sequential breaking of the invisible infrared beams by movements of the mouse was recorded, by the monitor, as ambulatory activity count. Mice were placed individually in the apparatus and the ambulatory count was recorded over 10 min, with the apparatus being cleaned with Virkon spray between subjects. Locomotor activity studies were performed one day a week, with mice being tested twice in the test day. Locomotor activity was first recorded between the hours of 08:00 and 11:00. In the afternoon of the same day, between the hours of 13:00 and 16:00, mice were injected with their daily dose of vehicle or THC. Twenty min later, they were placed in the apparatus and ambulatory activity count was recorded. The test carried out in the morning investigated the effect of chronic, daily administration of THC on locomotor activity while the test in the afternoon, 20 min post-THC or vehicle injection investigated the acute effect of the drug on activity.

### Whole gut transit

Whole gut transit is measured as the latency of a gavaged marker to appear in fecal pellets. Mice were acclimatized to individual, empty, plastic cages for 1 hour before being gavaged (using a 3 cm, 20 G gavaging needle) with 200 μl of Evans blue marker (5% Evans blue, 5% gum Arabic). Mice were returned to the empty test cages and the latency to the presence of blue fecal pellets was recorded. The acclimatization period is necessary as we have previously shown that upper gastrointestinal transit in mice is accelerated when mice are taken from their group-housed home cage and placed in an empty cage for 45 min [25].

### Microbiota

Fecal matter was collected prior to, and at the end of, the 4 week treatment period, in order to longitudinally measure the microbiota profile of each mouse prior to and following THC or vehicle treatment. Fecal samples were stored at -80°C until analysis. Microbial DNA was extracted from samples using FastDNA Spin Kit for Feces (MP Biomedicals, Lachine, QC) and quantified using PicoGreen DNA quantification kit (Invitrogen, Carlsbad, CA). Microbiota was quantified using qPCR as previously described [26]. Mice fed HFD excreted fecal pellets that were observed to be drier, smaller and fewer in number than those from the lean mice. As such it was not possible to collect enough fecal matter for microbiota analysis from all the DIO mice without removing them from their home cage for a prolonged period of time. This stressor may have had detrimental effects on their feeding behavior. As a result, the sample size for some groups is low.

### Statistics

Data are expressed as the mean ± standard error of the mean (s.e.m.) and analyzed using GraphPad Prism version 6.0 (GraphPad software, San Diego, CA.). Body weight and fat mass are expressed as the change from the measurement recorded before treatment with THC or vehicle began. Body weight change, fat mass change, energy intake, locomotor activity and whole gut transit data were analyzed using repeated measures ANOVA with time as the within-subject factor and THC treatment (+ or -) as the between-subject factor. When a significant time x treatment effect was found, Bonferroni’s, corrected for multiple comparisons, *post hoc* test was used to determine statistical differences at each time point. Microbiota data were analyzed using repeated measures ANOVA with time as the within-subject factor and THC treatment (+ or -) as the between-subject factor. When a significant time x treatment effect was found, a 1 way ANOVA, with Tukey’s *post hoc* test was carried out to determine statistical differences between all groups. *P*<0.05 was considered significant.

## Results

### Body weight, composition and energy intake

**Fig 1:**
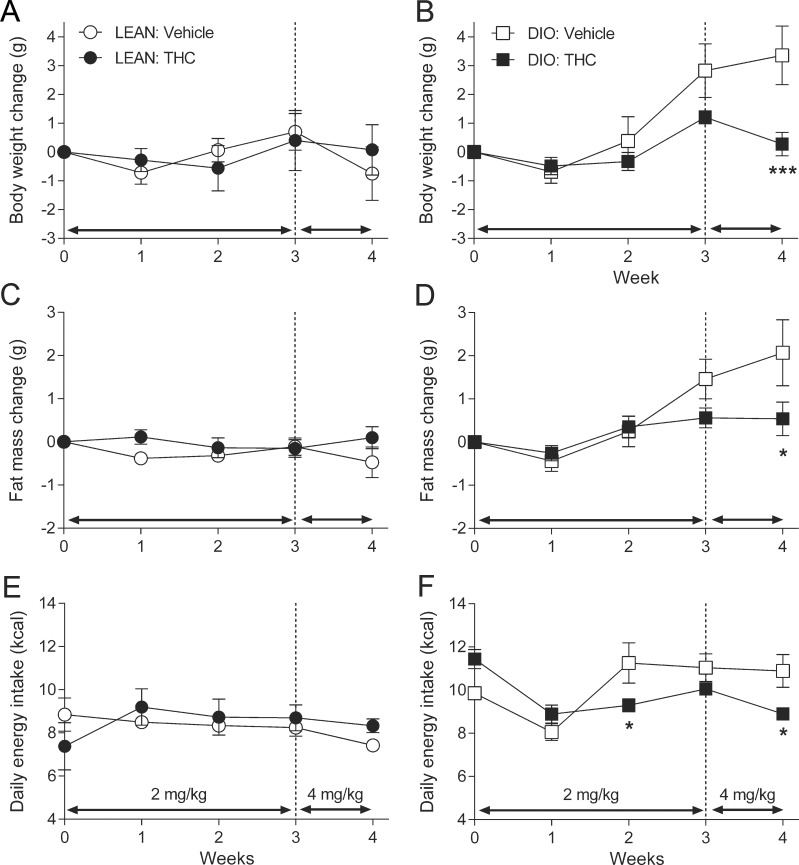
Chronic THC prevents high fat diet-induced change in body weight and fat mass. The effect of daily administration of Δ^9^-tetrahydrocannabinol (THC; 2 or 4 mg/kg) or vehicle on body weight change (**A**, **B**), fat mass change (**C**, **D**) and daily energy intake (**E**, **F**) in lean (**A**, **C**, **E**) or diet-induced obese (DIO) mice (**B**, **D**, **F**). Data points represent the mean ± s.e.m., *n* = 5–8. * *P* < 0.05 and *** *P* < 0.001 denote a significant difference from the vehicle control.

In lean mice there was no THC effect over time on body weight change (*F*(4, 36) = 0.90, *P* = 0.48), fat mass change (*F*(4, 36) = 1.61, *P* = 0.19) or daily energy intake (*F*(4, 36) = 2.16, *P* = 0.09) (Fig 1A, 1C and 1E). The interaction between time and treatment affected body weight change in DIO mice (*F*(4, 52) = 6.19, *P* < 0.001); with body weight change being significantly reduced by 4 mg/kg THC, but not 2 mg/kg THC, compared to vehicle (Fig 1B). There was a significant interaction between time and treatment effect on fat mass change in DIO mice (*F*(4, 52) = 3.35, *P* = 0.016) with fat mass change being significantly lower in 4 mg/kg THC treated mice than in vehicle treated mice (Fig 1D). There was an interaction effect of THC treatment over time on daily energy intake in DIO mice (*F*(4, 52) = 15.57, *P* < 0.001). The THC treated DIO mice consumed less calories in week 2 than the vehicle treated controls (Fig 1F) despite having comparable body weights at this point (THC group: 29.0 ± 0.5 g; Vehicle group: 30.1 ± 1.1 g). Daily energy intake was comparable between DIO vehicle-treated mice and mice treated with 2mg/kg for 3 weeks but 4 mg/kg THC-treated mice consumed fewer calories than control (Fig 1F).

### Locomotor activity

**Fig 2:**
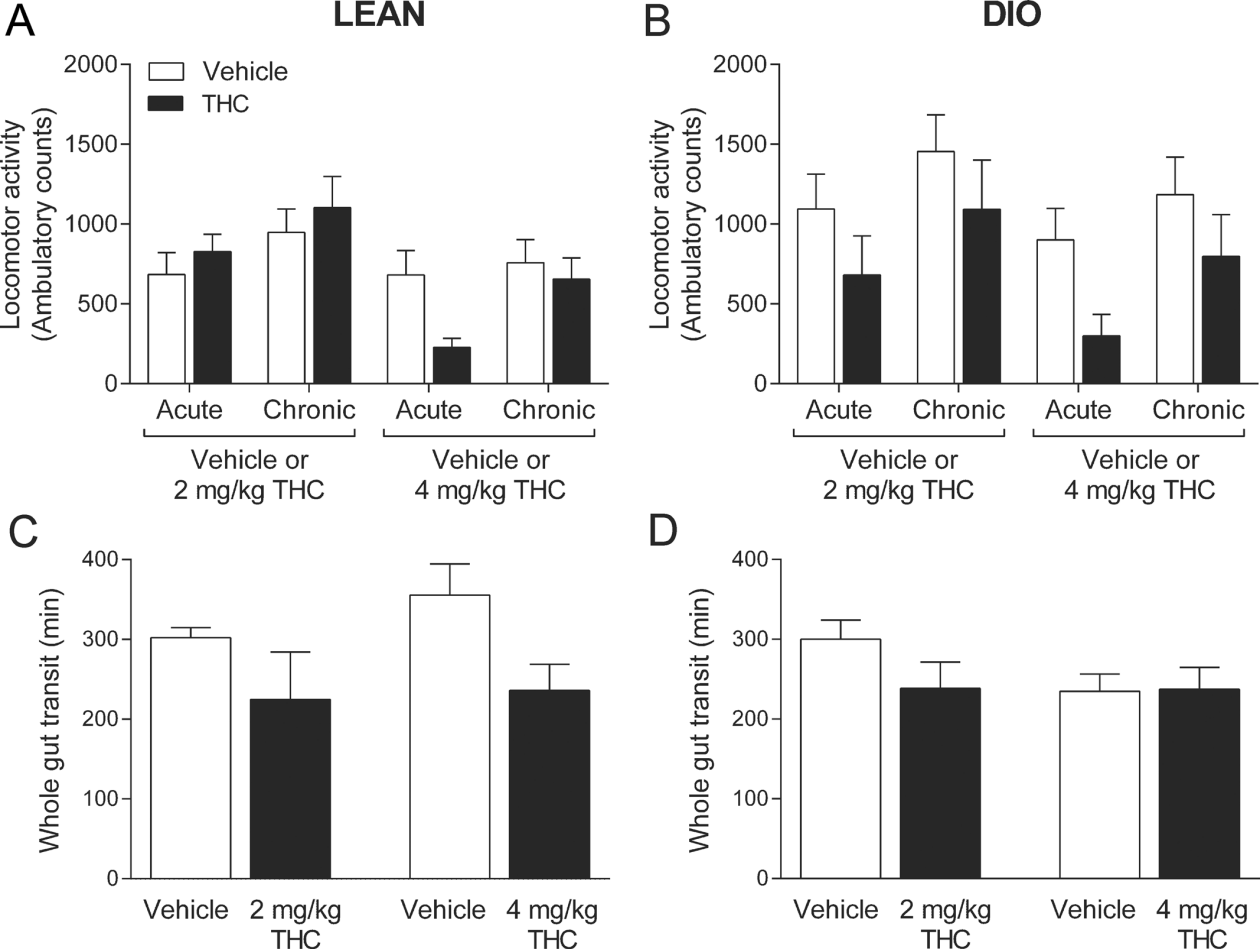
THC has no effect on locomotor activity or whole gut transit. The effect of daily administration of Δ^9^-tetrahydrocannabinol (THC) or vehicle on locomotor activity (**A**, **B**) in lean or diet-induced obese (DIO) mice recorded between 13:00–16:00, 20 mins after the daily treatment injection (Acute) or between 08:00–11:00 (Chronic) and on whole gut transit (**C, D**). Bars represent the mean ± s.e.m., *n* = 4–7.

There was a significant interaction effect between time and treatment for locomotor activity in lean mice (*F*(3, 24) = 4.07, *P* = 0.018). Bonferroni’s post hoc testing did not reveal a significant difference between locomotor activity in vehicle treated mice and THC treated mice at any point (Fig 2A) however, there was a non-significant trend for 4 mg/kg THC to acutely reduce locomotor activity in lean mice (Fig 2A). In DIO mice there was no effect of THC over time on locomotor activity (*F*(3, 36) = 0.48, *P* = 0.70; Fig 2B).

### Whole gut transit

There was no effect of THC treatment over time on whole gut transit in lean (*F*(1, 8) = 0.18, *P* = 0.68; Fig 2C) or DIO mice (*F*(1, 13) = 3.68, *P* = 0.08; Fig 2D).

### Microbiota

**Fig 3:**
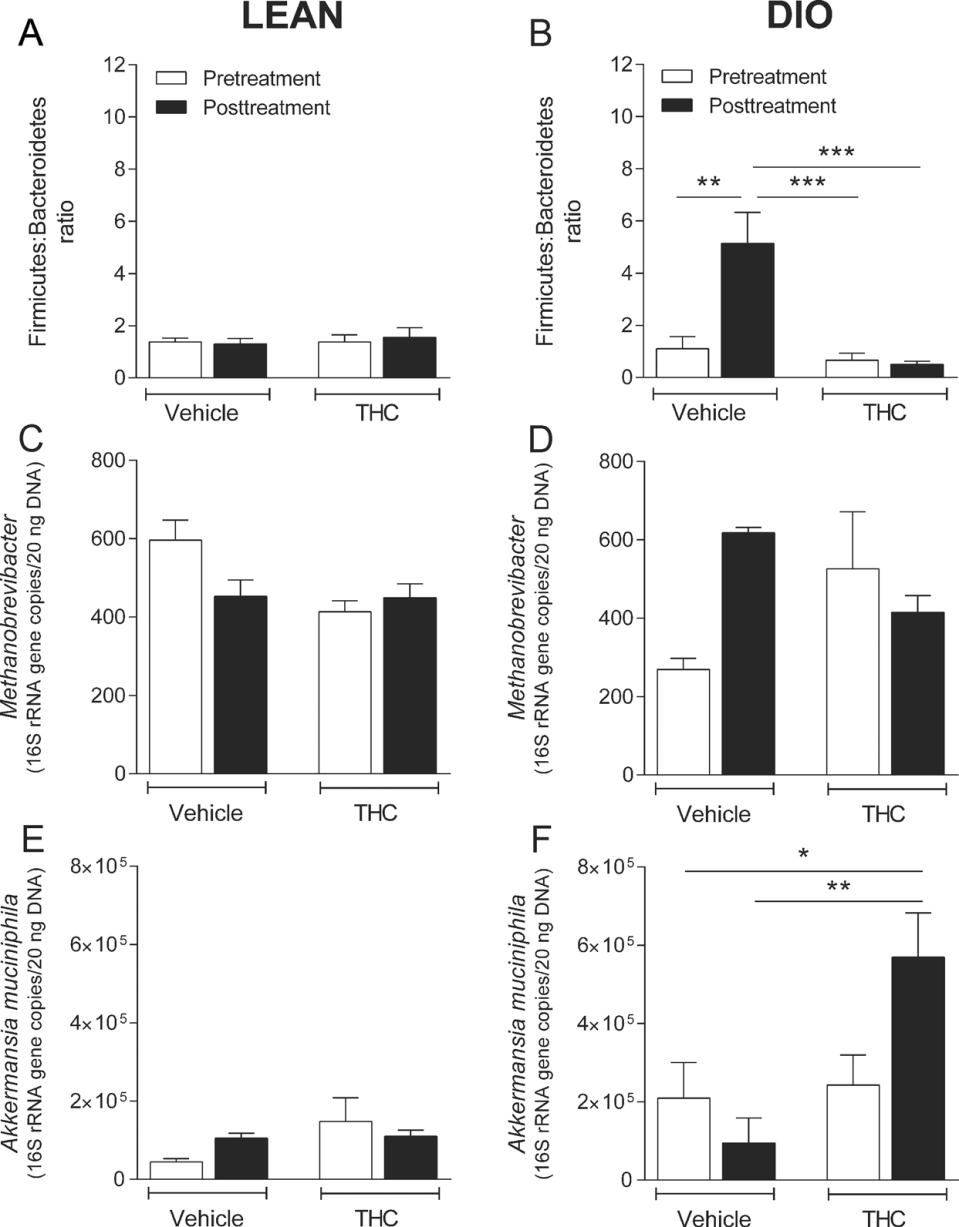
Chronic THC prevents some diet-induced changes in microbiota. Microbiota content in fecal matter of lean (**A, C, E**) or diet-induced obese (DIO; **B, D, F**) mice prior to (Pretreatment), and following (Posttreatment) chronic administration of Δ^9^-tetrahydrocannabinol (THC) or vehicle. Bars represent the mean ± s.e.m., *n* = 4–7. * *P*<0.05, ** *P*<0.01 and *** *P*<0.001 denotes a significant difference between the indicated groups.

In lean mice, fecal microbiota was not altered by chronic THC administration (Fig 3A, 3C and 3E and Table 1). In DIO mice there was a significant time x treatment interaction effect on Firmicutes:Bacteroidetes ratio (*F*(1, 8) = 8.25, *P* = 0.021); with the ratio being greater in vehicle-treated animals posttreatment than pretreatment, and being higher than the THC treated animals both pre and posttreatment. This pattern indicates that high-fat diet lead to an increase in the Firmicutes:Bacteroidetes ratio that was prevented by THC administration (Fig 3B). A similar trend was observed in *Methanobrevibacter* spp. but this difference did not reach significance (*F*(1, 7) = 5.01, *P* = 0.060. Fig 3D). Table 1 and Table 2 show the data of the Firmicutes (*Clostridium coccoides* (C-XIV), *C*. *leptum* (C-IV), *C*. cluster (C-XI), *C*. cluster (C-I) and *Roseburia* spp.) and Bacteroidetes (*Bacteroides/Prevotella* spp.) that make up the Firmicutes:Bacteroidetes ratio. THC increased *Akkermansia muciniphila* spp. abundance in DIO but not lean mice (Fig 3E and 3F). There was a time by treatment interaction effect on *Akkermansia muciniphila* in DIO animals (*F*(1, 8) = 9.37, *P* = 0.016) with a greater abundance of these bacteria in THC treated mice than in vehicle treated mice (Fig 3F).

**Table 1:** Microbiota content in fecal matter of lean mice prior to (Pretreatment, PreTx), and following (Posttreatment, PostTx) chronic administration of Δ^9^-tetrahydrocannabinol (THC) or vehicle.

	Vehicle		THC	
Microbial group	PreTx	PostTx	PreTx	PostTx
Total bacteria	19200 ± 1502	17810 ± 2564	14090 ± 1226	17720 ± 1868
*Bacteroides/Prevotella* spp.	4409 ± 300.5	3542 ± 1280	2573 ± 260.6	2979 ± 633.1
*Clostridium coccoides* (C-XIV)	3915 ± 214.4	1722 ± 172.1	2920 ± 428.8	3469 ± 595.2
*Clostridium leptum* (C-IV)	1980 ± 119.7 ^b^	463.6 ± 124.5 ^a^	414.7 ± 62.6 ^a^	527.3 ± 37.0 ^a^
*Clostridium* cluster (C-XI)	0.088 ± 0.018	0.145 ± 0.027	0.205 ± 0.035	0.166 ± 0.054
*Clostridium* cluster (C-I)	0.565 ±0.081	0.745 ± 0.198	0.420 ± 0.056	0.806 ± 0.224
*Roseburia* spp.	0.563 ± 0.131 ^b^	0.103 ± 0.032 ^a^	0.152 ± 0.056 ^a^	0.136 ± 0.025 ^a^

Values are mean ± s.e.m., 16S rRNA gene copies/20 ng DNA, n = 4–7 per group. Values were divided by 1000 so data shown in the table is 16S rRNA gene copies (10^3^)/20 ng DNA. Different superscript letters indicate significant differences between groups in a row. *P* < 0.05.

**Table 2:** Microbiota content in fecal matter of DIO mice prior to (Pretreatment, PreTx), and following (Posttreatment, PostTx) chronic administration of Δ^9^-tetrahydrocannabinol (THC) or vehicle.

	Vehicle		THC	
Microbial population	PreTx	PostTx	PreTx	PostTx
Total bacteria	18870 ± 1294	19580 ± 2565	22060 ± 1346	19980 ± 1081
*Bacteroides/Prevotella* spp.	6025 ± 1059	2196 ± 541.5	7831 ± 1023	5243 ± 799.5
*Clostridium coccoides* (C-XIV)	3621 ± 1730	8227 ± 936.9	3079 ± 1310	1691 ± 523.9
*Clostridium leptum* (C-IV)	1157 ± 388.5	741.4 ± 164.6	1215 ± 247.4	674.4 ± 105.3
*Clostridium* cluster (C-XI)	0.053 ± 0.018 ^a^	0.235 ± 0.025 ^b^	0.100 ± 0.019 ^a^	0.148 ± 0.030 ^a,b^
*Clostridium* cluster (C-I)	0.393 ± 0.266	0.603 ± 0.122	0.344 ± 0.086	0.366 ± 0.093
*Roseburia* spp.	0.284 ± 0.187	3.045 ± 0.955	0.397 ± 0.13	0.073 ± 0.026

Values are mean ± s.e.m., 16S rRNA gene copies/20 ng DNA, n = 4–7 per group. Values were divided by 1000 so data shown in the table is 16S rRNA gene copies (10^3^)/20 ng DNA. Different superscript letters indicate significant differences between groups in a row. *P* < 0.05.

## Discussion

We present data showing that chronic administration of the CB_1_/CB_2_ receptor partial agonist, THC, prevents weight gain in DIO mice. Furthermore, we show evidence that DIO-mediated modifications in gut microbiota are prevented in chronically THC treated mice.

Prevention of weight gain in THC treated mice, fed a high fat diet was associated with a reduction in energy intake. The effect on body weight was due mainly to an inhibition of increased fat mass. THC prevented high fat diet-induced weight gain at a dose which had no significant effect on locomotor activity and which had no effect on body weight in lean mice. THC has sedative properties which can prevent access to the food source. Chronic THC administration has been shown to reduce body weight in lean rodents but at doses that concurrently inhibited locomotor activity [27] or in studies where sedation was not measured [28]. In the current study there was a trend for locomotor activity to be reduced 20 min after THC administration in DIO mice. This trend was not observed 16 h later suggesting that this mild effect was transient. Similarly, there was a trend for locomotor activity to be acutely suppressed by THC in lean animals yet energy intake was not inhibited in these animals, suggesting that in DIO mice the mild and transient effect of THC on locomotor activity is not responsible for hypophagia and prevention of weight gain. THC-induced hypophagia observed in DIO mice is unlikely to be due to general malaise as there was no effect of THC on energy intake in lean animals. It has been postulated that as a partial CB_1_/CB_2_ agonist that therefore does not elicit maximal stimulation of the receptor, THC could block endogenous, full agonists from binding to CB_1_ receptors in situations of high endocannabinoid tone, as seen in obesity [21]. The current study confirms that chronic THC prevents DIO and future investigation of its pharmacological mechanism of action is warranted.

Chronic THC failed to modify whole gut transit in DIO mice suggesting that the effects on body weight, energy intake and gut microbiota (see below) were not due to altered rate of the passage of nutrients in the gut. Interestingly, in lean mice chronic THC showed a trend to reduce whole gut transit, shortening the time taken for the blue marker to be defecated. Cannabinoid receptor CB_1_/CB_2_ full agonists such as WIN55,212–2 have well characterized inhibitory actions on gastric emptying and gastrointestinal transit in rodents [29–32]. THC inhibited gastric motor activity in rats via CB_1_ [33] in a COX dependent manner [34] while Dronabinol® (synthetic THC) had no effect on small intestinal transit or colonic transit in healthy humans [35]. In contrast to the aforementioned studies, the current study did not investigate the effect of THC immediately after its administration, rather the chronic effect of THC was being examined, such that whole gut transit studies were carried out through the morning and early afternoon, before the daily injection of THC in the late afternoon. Thus it is unlikely that THC-induced prevention of weight gain in these mice is due to altered gastrointestinal motility.

We show that DIO-induced changes in the microbiota are prevented in mice administered chronic THC. The gut microbiota modifies endocannabinoid signaling in obesity; increasing gut permeability and contributing to obesity-associated low-grade inflammation and influencing adipogenesis [20]. The current study demonstrates that THC-induced prevention of weight gain is associated with modifications in microbiota. Whether or not the effect of the gut microbiota on endocannabinoid signaling is reciprocal such that the changes in endocannabinoid tone in obesity influence the composition of gut microbiota warrants a much more detailed investigation.

Whether our findings show that THC caused modifications in gut microbiota leading to the prevention of weight gain in high fat feeding or whether the altered microbiota was a consequence of THC-induced prevention of weight gain remains to be elucidated. A paired-feeding study is warranted to investigate this. However, evidence shows that microbiota changes in the reversal of obesity may not be a result of weight loss per se [36] and the mechanism by which body weight is modified may play a role.

The microbiota profile in obesity is classically characterized by increased abundance of microbial species in the division *Firmicutes* and a reduced abundance of those in *Bacteroidetes*; levels of which are restored by weight loss due to calorie restricted diet [37] and prebiotic fiber supplementation [38]. In the current study, an increased Firmicutes:Bacteroidetes ratio was similarly observed in chronic THC treated DIO mice. It has been shown that the differences in microbiota after bariatric surgery-induced weight loss are not specifically due to modified BMI but are due to the surgery itself [36]. Microbiota changes following weight loss are not ubiquitous. Weight loss through bariatric surgery induces alterations in microbiota that are different to those observed in weight loss due to changes in diet [36]. Microbiota changes observed in weight loss through dietary intervention are characterized by shifting the Firmicutes:Bacteroidetes ratio [37] whereas weight loss following bariatric surgery was associated with an increase in Proteobacteria [36]. This suggests that THC may be exerting unique effects on the microbiota to prevent weight gain in the current study and this exciting prospect requires a further investigation.

In the current study we observed an increase in *Akkermansia muciniphila* abundance in DIO mice treated with chronic THC. *A*. *muciniphila* is a mucin degrading bacteria involved in the regulation of mucus in the gastrointestinal tract [39] and its abundance has been shown to increase following weight loss induced by oligofructose prebiotic consumption [40]. Everard *et*. *al*. [40] further demonstrated that *A*. *muciniphila* controls fat storage and adipose tissue metabolism leading to weight loss. It is possible that the actions of *A*. *muciniphila* on adipose tissue may play a role in mediating the actions of THC to prevent DIO.

In conclusion, we present data showing the CB_1_/CB_2_ receptor partial agonist THC, induces hypophagia and prevents weight gain in obesity and suggest these actions may be mediated in part by modifications of the gut microbiota.
